# Chemical Space Exploration with Active Learning and Alchemical Free Energies

**DOI:** 10.1021/acs.jctc.2c00752

**Published:** 2022-09-23

**Authors:** Yuriy Khalak, Gary Tresadern, David F. Hahn, Bert L. de Groot, Vytautas Gapsys

**Affiliations:** † Computational Biomolecular Dynamics Group, Department of Theoretical and Computational Biophysics, Max Planck Institute for Multidisciplinary Sciences, Am Fassberg 11, D-37077 Göttingen, Germany; ‡ Computational Chemistry, Janssen Research & Development, Janssen Pharmaceutica N. V., Turnhoutseweg 30, 2340 Beerse, Belgium

## Abstract

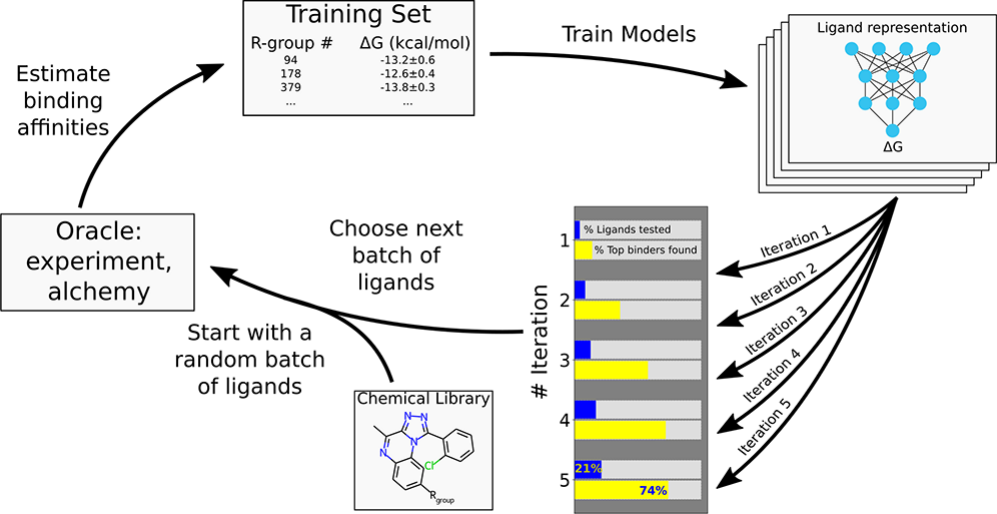

Drug discovery can be thought of as a search for a needle in a haystack: searching through a large chemical space for the most active compounds. Computational techniques can narrow the search space for experimental follow up, but even they become unaffordable when evaluating large numbers of molecules. Therefore, machine learning (ML) strategies are being developed as computationally cheaper complementary techniques for navigating and triaging large chemical libraries. Here, we explore how an active learning protocol can be combined with first-principles based alchemical free energy calculations to identify high affinity phosphodiesterase 2 (PDE2) inhibitors. We first calibrate the procedure using a set of experimentally characterized PDE2 binders. The optimized protocol is then used prospectively on a large chemical library to navigate toward potent inhibitors. In the active learning cycle, at every iteration a small fraction of compounds is probed by alchemical calculations and the obtained affinities are used to train ML models. With successive rounds, high affinity binders are identified by explicitly evaluating only a small subset of compounds in a large chemical library, thus providing an efficient protocol that robustly identifies a large fraction of true positives.

## 1 Introduction

The endeavor of drug discovery can be viewed as chemical space exploration with an aim to simultaneously optimize multiple properties, e.g., ligand binding affinity to the target, synthetic accessibility, and toxicity. As this search space is vast, estimates go up to 10^60^ drug-like compounds,^1^*in vitro* and *in vivo* library screens are able to cover only a minor fraction of the possible solutions. To this end, computational chemoinformatic and physics-based approaches have been employed to increase the reach of the chemical space explorations.

Over the recent years, with the advent of artificial intelligence (AI) methodology, machine learning (ML) approaches saw a rapid adoption in drug discovery. A lot of research has been devoted to constructing artificial neural networks capable of exploring chemical space to suggest novel drug-like candidate molecules for further screens.^2−4^ Deep learning methods have also been successfully applied to predict ligand molecular properties and establishing QSAR models.^5^ Establishing structure activity relationships requires accurate prediction of the ligand binding affinity to the target protein and remains a key challenge for computational chemists. Active learning (AL) approaches present a promising pathway to this goal.

The AL methodology comprises an iterative approach where the machine learning models suggest new compounds for an oracle (experimental measurement or a computational predictor) to evaluate. These compounds and their scores are then incorporated back into the training set for further improvement of the models.

For example, machine learned models have been used to predict results of free energy calculations^6−8^ or molecular docking.^9^ Subsequently, a fraction of the compounds was selected for calculations in the next iteration. Feeding the results of the calculations back into the ML training and iterating the process in a loop allowed to efficiently screen through a large chemical library.

Alchemical free energy calculations^10−12^ based on first principle statistical mechanics may serve as an optimal input for such AL applications. While computationally demanding, nowadays these calculations are readily accessible even at large scale: the predictions for hundreds to thousands of ligands can be obtained in a matter of days.^13,14^ Also, the accuracy of alchemical predictions draws close to the experimental measurements.^15−18^ Therefore, using these calculations as an oracle to construct ML models could allow describing binding affinities of large chemical libraries with high accuracy, while only a small fraction of the library needs to be evaluated with the computationally expensive alchemical method.

In the current work, we apply AL approaches to the lead optimization step of drug discovery. In the first part of the study we retrospectively analyzed a large set of phosphodiesterase 2 (PDE2) inhibitors for which experimentally measured binding affinities were readily available. We explored the optimization of the learning process with respect to the ligand selection procedure for the free energy calculations, the molecule encoding for ML, and the hyper-parameter tuning of the ML models.

Having established the optimal set of parameters to efficiently navigate in this chemical subspace, we proceeded with a prospective search for potent PDE2 inhibitors. We generated an *in silico* compound library and navigated using an active learning protocol based on the alchemical free energy calculation oracle. Lead optimization performed this way recovered multiple ligands with strong computed binding affinities, with only a small fraction of compounds screened by computationally costly alchemical calculations.

## 2 Methods

### 2.1 Generating Ligand Binding Poses

For the retrospective ligand library, which spans multiple different scaffolds, multiple aligned crystal structures with bound inhibitors were considered for use as reference structures for starting pose generation: 4D08,^19^ 4D09,^19^ 4HTX,^20^ 6CYD,^21^ 6EZF,^22^ as well as 13 unpublished structures shared with us by Janssen Research & Development. For each ligand in the retrospectively analyzed library (Part I) the inhibitor with the highest Dice similarity^23^ based on the RDKit topological fingerprint^24^ was used as the reference. For the prospective investigation in Part II, the generated ligand library shared a core with the inhibitor from the 4D09 crystal structure;^19^ thus, 4D09 coordinates were used as a reference for the generation of binding poses for each ligand in the library.

Afterward, coordinates of the largest substructure matches between each ligand and its reference were constrained to the same coordinates as in the crystal structure. The remaining atoms initial guesses were assigned via constrained embedding following the ETKDG algorithm^25^ as implemented in RDKit.^24^ This approach was not always able to respect the constrained positions of the common substructures and would return a different outcome depending on the initial random seed. One hundred of such structures were constructed for each ligand, and the one with the smallest RMSD to the reference was selected.

Ligand binding poses were then refined by molecular dynamics simulations in a vacuum. Here, the 6EZF^22^ structure was used for the retrospectively analyzed ligand library (Part I), and the 4D09 crystal structure^19^ was used for the prospective library (Part II). First, a hybrid topology between the reference inhibitor and each ligand was constructed with pmx,^26^ and the coordinates of the largest common substructure were restrained with a force constant of 9000 kJ/(mol nm^2^). Next, the energy of the protein and reference inhibitor system was minimized. Finally, the reference inhibitor was morphed into the ligand following the hybrid topology while simultaneously lowering the temperature from 298 to 0 K in a 10 ps simulation. Ligand coordinates from the final frame were treated as the binding pose and used to construct both the ligand representations for machine learning and as starting ligand coordinates for the relative binding free energy calculations.

### 2.2 Ligand Representations and Feature Engineering

Machine learning of ligand properties requires a consistent, fixed-size vector representation for each ligand. These are typically composed of molecular fingerprints and/or constitutional, topological, geometric, thermodynamic, and electronic features of the molecule, an overview of which can be found elsewhere.^27^ Here, we explored several representations to encode the ligand library.

The first and most complex representation was built from all the features we could compute directly with RDKit^24^ from ligand topologies and 3D coordinates. Hence, we refer to this representation as 2D_3D. These features include constitutional, electrotopological, and molecular surface area descriptors as well as multiple well-established molecular fingerprints. A more detailed breakdown is shown in Table S3.

Another representation was based on MedusaNet^28^ and allows for encoding the three-dimensional shape and orientation of a ligand in the active site. For this representation we split the binding site into a grid of cubic voxels with 2 Å edge length and counted the number of ligand atoms of each chemical element in each voxel resulting in a sparse 4-dimensional tensor. Unlike in the original MedusaNet paper,^28^ which dealt with convolutional neural networks, we used a one-dimensional representation of the tensor, as we work with linear layers instead. We refer to this representation as atom-hot, as it is similar to one-hot encoding used to label training data for classifiers in machine learning, except for multiple atoms being able to occupy the same voxel. Additionally a modified version, called atom-hot-surf, was probed. This representation only considered voxels on the van der Waals surface of the binding pocket.

The rest of the representations encoded protein ligand interactions. The PLEC fingerprints^29^ were constructed by means of the Open Drug Discovery Toolkit v0.7^30^ to represent the number and type of contacts between the ligand and each protein residue from the 4D09 crystal structure. Additionally, we also used a pair of representations composed of both electrostatic and van der Waals interaction energies between the ligand and each protein residue with at least one atom within 1.5 nm of any ligand in the library. Both were computed with Gromacs 2021.1,^31^ the Amber99SB*-ILDN force field^32−34^ for the protein, and the GAFF 1.9 force field^35^ for ligands. The energies were evaluated at two different cutoff values: 1.1 nm for the MDenerg representation and 5.1 nm for MDenerg-LR representation.

Finally, in the first three iterations for the prospectively analyzed data set (Part II), R-group-only versions of all of the above representations were also used in addition to the complete ligand ones described above. In these representations, features that were impossible to calculate for all ligands in the library given the much smaller structures, like parts of the GETAWAY fingerprint, were dropped from the respective representations.

### 2.3 Ligand Selection Strategies

The character of chemical space exploration can be altered by modifying the selection strategy of ligands to be presented for an evaluation by the oracle. We have probed the following strategies to select a batch of 100 ligands at every iteration:

- *random* selection of ligands;
- *greedy* selects only the top predicted binders at every iteration step;
- *narrowing* strategy combines broad selection in the first 3 iterations with the subsequent switch to *greedy* approach. For the first iterations, several models are trained, each using different sets of the previously described ligand descriptors and the 5 models with the lowest cross-validation RMSE are identified. From each of those models, the 20 best predicted binders are then selected;
- *uncertain* strategy selects the ligands for which the prediction uncertainty is the largest;
- *mixed* strategy first identifies the 300 ligands with the strongest predicted binding affinity (three times more than with *greedy* selection), and then selects the 100 ligands with the most uncertain predictions among them.

In all the cases, initialization of the models (iteration 0) was based on the weighted random selection. Namely, the ligands were selected with the probability inversely proportional to the number of similar ligands in the data set. Ligands were considered similar if after a t-SNE embedding^36^ they fell within the same bin of a 2D histogram (the square bins of the 2D histogram had a side length of one unit in the t-SNE space). The embedding was constructed from the ligands’ 2D features (constitutional and graph descriptors as well as MACCS^37^ and BCUT2D^38^ fingerprints) using the full ligands for the retrospective library and only the R-groups for the prospective library.

### 2.4 The Oracle: Alchemical Free Energy Calculations

Free energy calculations were used to generate training targets in the prospective data set (Part II). These calculations were based on the molecular dynamics simulations relying on the nonequilibrium free energy calculation protocol^10,17^ based on Crooks’ Fluctuation Theorem.^39^ The perturbation maps were constructed using a star shaped map topology,^40^ where a single ligand with the experimentally measured binding affinity and common scaffold with the rest of the compounds was used as a reference for all perturbations.

All the ligands were considered in their neutral form using a single tautomer, as generated by RDKit. First, the ligands were parametrized with GAFF 1.81 using ACPYPE^41^ and AnteChamber^42^ with AM1-BCC charges^43^ and off-site charges for halogen atoms.^44^ A hybrid topology was then built for each evaluated ligand against the reference ligand with pmx.^26^

Solutes for the two legs of the thermodynamic cycle were assembled. One leg of the cycle contained the protein (parametrized by the Amber99sb*ILDN^32−34^ force field) from the 4d09 crystal structure,^19^ the crystallographic waters, and the hybrid ligands positioned according to their previously determined binding poses. The other branch contained only the hybrid ligands. The structures were then solvated with TIP3P^45^ water and 0.15 M sodium and chloride ions parametrized by Joung and Cheatham^46^ in a dodecahedral simulation box with 1.5 Å padding. All subsequent simulations were carried out with Gromacs 2021.6^31^ with a 2 fs integration time step.

Prior to production runs, energy minimization and, for the protein and ligand leg of the cycle, a short 50 ps NVT simulation were performed. During these runs the solute heavy atoms were position restrained with a force constant of 1000 kJ/(mol nm^2^). Subsequently, 6 ns equilibrium simulations were performed in an NPT ensemble. The temperature was kept at 298 K with the velocity rescaling thermostat^47^ with a time constant of 2 ps. One bar pressure was retained with the Parrinello–Rahman barostat^48^ with a time constant of 5 ps. Electrostatic interactions were handled via Smooth Particle Mesh Ewald^49,50^ with 1.1 nm real space cutoff. Van der Waals interactions were smoothly switched off from 1.0 to 1.1 nm. Isotropic corrections for both energy and pressure due to long-range dispersion^51^ were applied. All bond lengths were constrained via the LINCS algorithm.^52^

From the generated trajectories the first 2.25 ns were discarded, and the remaining simulation frames were used to initialize alchemical nonequilibrium transitions between the two end states: 80 transitions in each of the two directions. 50 ps long nonequilibrium alchemical transitions were started from each frame, and the work needed to perform the transition was recorded. Relative binding free energies were calculated from the bidirectional work distributions using a maximum-likelihood estimator^53^ implemented in pmx.^26^ The whole equilibration-transitions-analysis protocol was repeated five times for each evaluated ligand and the mean and standard error of the five repeats were taken as the relative binding free energy and associated uncertainty. To obtain the absolute binding free energy for use in the training set, the relative free energy was combined with the experimentally known absolute binding free energy of the reference ligand.

### 2.5 Model Architecture

Regression models for free energy prediction were ensemble models^54^ of multilayer perceptrons with ReLU^55^ activation functions. Each individual perceptron was trained on a 5-fold split of the training data, each leaving out one fold for cross-validation, and was initialized with different weights and biases. Each produced different predictions for ligands in regions of chemical space where insufficient training data was available. Averaging over the predictions of independent models allowed us to recover not only more precise values but also more accurate ones.^56^ Final predictions in most of this work came from means of 5 models with standard errors used for uncertainties. However, iterations four and five of active learning on the prospective library further expand the ensembles to average the final prediction over five repeats of the above cross-validation training procedure, leading to averaging over 25 individual models in total for these iterations.

Varying network depths and layer widths were probed. Preliminary hyper-parameter optimization of these values was carried out on the retrospectively analyzed data set in Part I. The resulting values were used for the first three iterations of active learning on the prospective data set in Part II. In iteration 4, hyper-parameters were reoptimized and feature selection was performed by selecting the best combination of previously described ligand encodings to use for the available training data. The combination of 2D_3D descriptors and PLEC fingerprints performed the best. In addition, for this iteration feature selection was performed by discarding the features whose mean importance determined by Integrated Gradients^57^ was under 0.02. Subsequent iterations used the full 2D_3D ligand representation without further feature selection. The details of the meta-parameters used with the prospective data set are in Table S1. Active learning on the retrospective data set reused many of the hyper-parameter values from the corresponding iterations of the prospective case (Table S2).

Distributions of the input feature values were normalized to zero mean and unit variance for each feature independently. Similar normalization of the training free energies was also attempted. However, better model accuracies were observed with manually optimized scaling and bias values (Table S1).

### 2.6 Model Training

Training of models was done for 2000 or 20 000 epochs with L1 loss function (absolute error between the prediction and training data). The stochastic gradient descent optimizer^58^ with a momentum of 0.9 and batches of up to 500 training ligands were used. An exponentially decaying learning rate of 0.005 × 0.1^epoch/10000^ was employed. Inverse frequency weighting was used to weigh the loss from individual training examples based on a Gaussian kernel distribution of the training free energies to remove bias due to overrepresentation of medium and high affinity ligands (Figure 3C). Early stopping based on cross-validation loss was used to limit overtraining.

### 2.7 Ligand Library Construction

The ligand library for the prospective PDE2 inhibitor study in the Part II of the manuscript was constructed around a modified core from the4D09PDB entry.^19^ A manual examination of the data set explored in Part I revealed that chlorination of the cyclohexene ring at different positions and addition of a methyl or a difluoromethyl to the tricycle led to better binding affinities, and a single combination of these features was chosen as the core of the current library; chemical space exploration was restricted to the remaining R-group (Figure 4A). The various R-groups were built up from fragments present in the data set from Part I to increase the likelihood of synthetic accessibility of the ligands.

Such fragments were obtained by removing the common cores from each ligand series in the data set and decomposing the remainders into chemical groups with the BRICS algorithm^59^ as implemented in the RDKit version 2021.03.3^24^ while keeping track of the atoms bonding to the cores and to other fragments. This resulted in two groups of fragments: linkers (Figure S6), which directly bond to the cores, and termini (Figure 4A), which bond to the linkers. The library R-groups were assembled by attaching each linker to the core by the same atom it would attach to the cores in the original data set from Part I. Different numbers and combinations of termini were then added to the designated linker’s atoms.

## 3 Results

### 3.1 Active Learning Cycle

**Figure 1:**
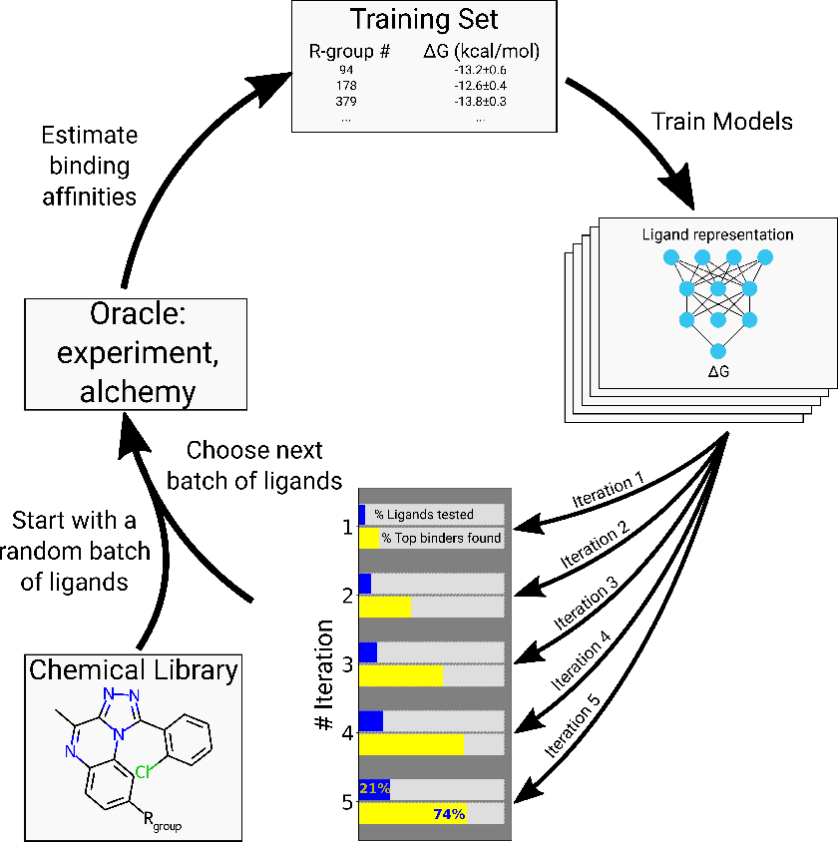
Active learning scheme. Models are trained to reproduce free energies obtained experimentally or computed by MD. At each iteration a batch of ligands is selected to be added to the training set based on their predicted free energies according to the previous iteration’s models. Iterative training of the models with an increasing training data set improves prediction accuracy: most of the top binders are identified by probing only a small part of the whole chemical library.

Throughout the work we employ an active learning cycle, as depicted in Figure 1, to explore chemical space of PDE2 inhibitors. In the AL cycle, the process is started by assembling a chemical library of interest and initializing the procedure by a weighted random selection of a batch of compounds for the first iteration to ensure ligand diversity. The binding affinities of the selected ligands are evaluated in an alchemical free energy calculation procedure. These ligands together with the obtained affinity estimates form a training set for machine learning (ML) models, which, in turn, predict binding affinities for all the ligands in the chemical library. In the next iteration, another set of compounds is selected, and the same steps of the cycle are repeated. This way, the training set keeps increasing, thus improving the accuracy of the ML predictions. Most of the compounds with the highest binding affinity are identified in a small number of iterations of the cycle. In the process only a small fraction of the chemical library is evaluated explicitly with the computationally expensive physics-based approach, while affinities for the rest of the ligands are predicted by the ML model.

To optimize the parameters of the active learning protocol, we start with applying this scheme on a large collection of PDE2 inhibitors for which the binding affinities have been measured experimentally (Part I of the study). This allows us to replace the computationally expensive alchemical free energy calculation step with a simple lookup table of measured affinities. In doing so, in Part I of the study we are able to explore various approaches for ligand encoding, their selection procedures, and the effects on the ML predictions.

In Part II of the study, we apply the active learning cycle prospectively, now using alchemical free energy calculations to guide the model training.

### 3.2 Part I: Protocol Evaluation on a Retrospective Data Set

In the first part of the investigation, we explored the efficiency and convergence of the active learning protocols on a data set of PDE2 inhibitors with experimentally measured binding affinities. The collection of 2351 ligands interacting with PDE2 has been assembled in Janssen Pharmaceutica from the corresponding drug discovery project. This ligand set presents a convenient case for probing different versions of model building protocols, directly based on experimentally measured Δ*G* values, rather than relying on computational methods. This way, the oracle in Figure 1 is represented by the experimentally obtained affinities.

We started by generating binding poses for the ligands relying on the 6EZF crystal structure of PDE2, followed by encoding ligand representations for machine learning. As this collection contains molecules with a variety of chemical scaffolds, ligands could not be uniquely described solely by R-groups attached to a single scaffold. Hence, only representations involving features of complete ligands were used.

#### 3.2.1 Ligand Representation

**Figure 2:**
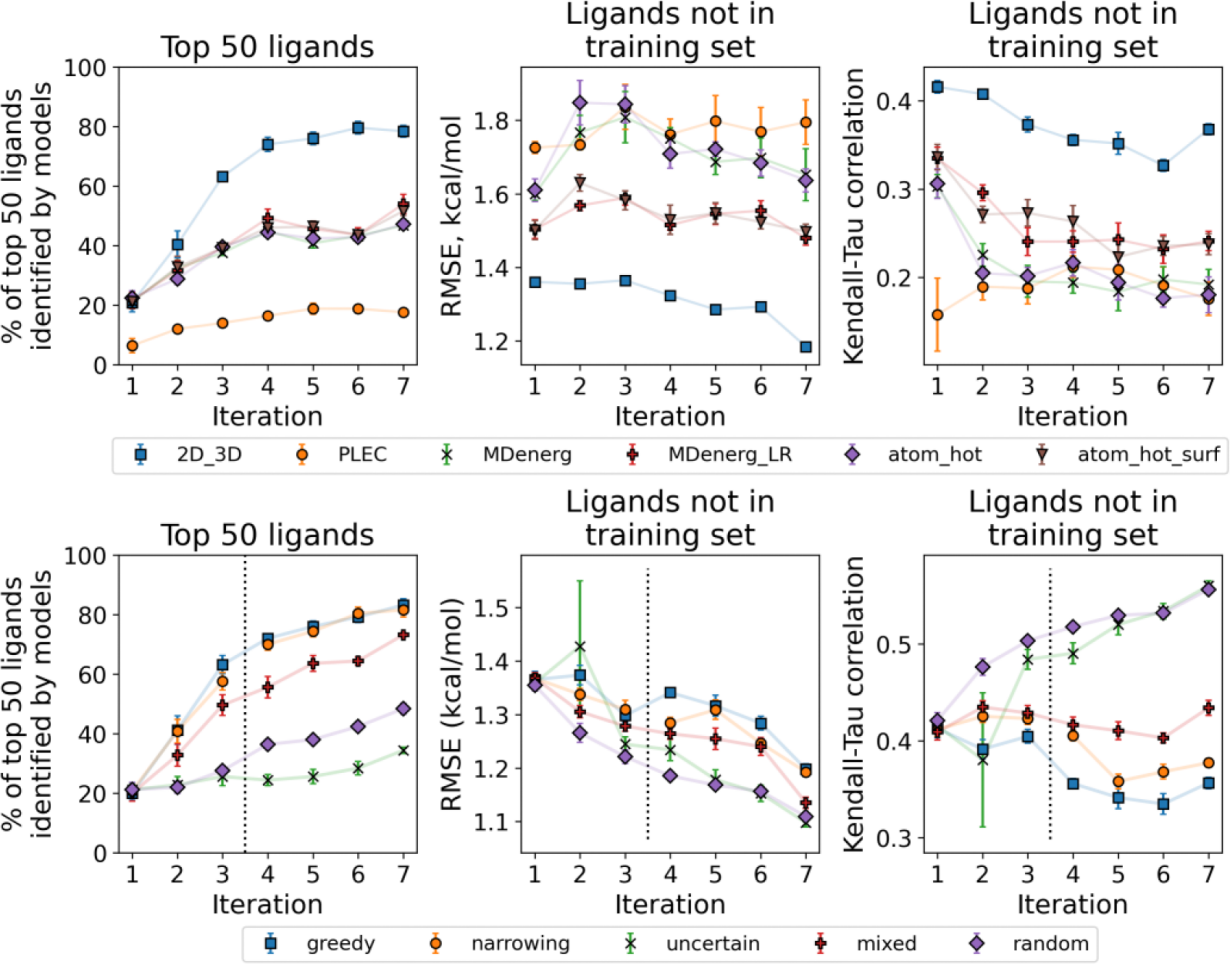
Accuracy comparisons of different ligand representations (top) and ligand selection strategies (bottom). Representations are evaluated with the *greedy* selection scheme, while the selection strategies with the 2D_3D representation composed of all the features supported by RDKit.

Both ligand representation and their selection protocol are essential components for the efficiency and accuracy of the active learning protocol in Figure 1. First, we evaluated the effectiveness of different ligand representations by encoding the ligand library with diverse 2D and 3D ligand descriptors (2D_3D), ligand–protein interaction fingerprints (PLEC), interaction energies from molecular mechanics force fields (MDenerg, MDenerg_LR), and grid-based ligand representations (atom_hot and atom_hot_surf) (Figure 2, top row). These ligand representations are described in more detail in the Methods section.

Relying on a simple *greedy* selection rule, we performed the cycles of active learning protocol choosing 100 ligands at a time and using the experimentally measured Δ*G* values for reference. The 2D_3D representation composed of all the chemoinformatic features supported by RDKit^24^ consistently outperformed the physics-based representations (MDenerg, MDenerg_LR, and PLEC) both in model accuracy and in the rate at which strongly binding ligands were identified. The PLEC fingerprint representation was finding strong binders much slower than the others. Meanwhile, the 2D_3D representation yielded the same top ligands more consistently than other representations (Figure S1).

#### 3.2.2 Ligand Selection Strategy

Having identified the 2D_3D descriptor representation as the most robust molecular encoding, we further investigated the performance of ligand selection strategies. In the above analysis we used the *greedy* strategy of selecting the strongest binders predicted at every iteration. While this leads to rapid improvement of binding affinities, it runs the risk of getting trapped in the first local minimum that is found. To mitigate this risk, we developed the *narrowing* strategy, where in the first three iterations we instead focus on broadening the scope of exploration in the chemical library and in the later iterations switch to the *greedy* selection mode. For these first three iterations, we train separate models for all the ligand representations discussed above as well as binned variants of MDenerg and MDenerg_LR, select the five that have the best internal cross-validation RMSE, and use the top 20 ligands predicted by each of them. If multiple representations yield the same ligand in their top 20s, we use the next best ligand from one of the representations, making sure that we select 100 unique ligands for the next iteration. After the third iteration we switch to the *greedy* approach with the 2D_3D representation to descend deeper into the best local minimum found so far. All in all, this strategy performs similarly to the greedy approach and finds the most potent binders in the library at a comparable rate (Figure 2 bottom row).

In addition to the *greedy* and *narrowing* protocols, we probed three more strategies: *uncertain*, *mixed*, and *random*, all shown in the lower panel of Figure 2.

One might expect that *random* selection of ligands to construct the ML models would yield suboptimal predictions, yet this depends on the particular objective that is set for exploring chemical space. The *random* approach describes well the general features of the chemical library (low RMSE, high correlation). This comes as a consequence of arbitrarily selecting ligands of diverse chemistry for model training. However, such a seemingly good performance of the *random* selection comes with a shortcoming: few potent compounds are being selected (low percentage of Top 50 ligands). Thus, *random* sampling of the chemical space could be used to obtain a general description of the library, but for ligand affinity optimization a different strategy might be preferred.

The *uncertain* strategy prioritizes selection of the predictions for which the model showed the largest uncertainty. Here, we model this uncertainty as standard deviation in predictions of 5 ensemble models trained on the same data with different random starting weights. Similar to *random* selection, this selection strategy places no priority on finding strong binders, yet both the rate of their discovery and the accuracy of their predicted free energies are worse than in the *random* selection of ligands.

We also explored a *mixed* strategy first proposed by Yang et al.^9^ This strategy selects the most uncertain ligands among a larger number of the strongest predicted binders. While the *mixed* strategy outperforms *random* ligand selection, in contrast to the finding of Yang et al., it identifies desirable ligands slower than the *narrowing* and *greedy* approaches. Here, we used a 3:1 ratio of the number of selected predicted strongest binding ligands to the number of selected most uncertain ones among them. In the original work by Yang et al. the ratio was 50:1, possibly explaining the observed difference in performance. However, a ratio this large was impractical in our case given the limited size of our data set of 2351 ligands only. In practice, the performance of this selection approach can be tuned by changing the above ratio, becoming equivalent to the *greedy* strategy with a 1:1 ratio, i.e., selecting only the strongest binders, and equivalent to the *uncertain* strategy when the identified strongest binders cover the whole data set. However, such tuning is impractical in a real prospective study, as it requires rerunning the AL protocol multiple times and finding reference free energies for all the discovered ligands in each repeat to identify the optimal ratio.

As the protocol progresses and more ligands are added to the training sets, all selection strategies result in better agreement with the predictions from the final iteration, but the *uncertain* strategy improves correlations the fastest (Figure S2). Overall, the *greedy*, *narrowing*, and *mixed* approaches are able to quickly locate the best binding ligands. The *greedy* and *narrowing* approaches do this faster and more consistently identify the same high affinity binders (Figure S3). However, the Kendall’s rank correlation between the predicted and experimental binding free energies is low for all the selection methods besides the uncertainty-driven one (Figure S4). This results in large numbers of ligands with lower experimental affinities being selected for evaluation and inclusion into the training data set at each iteration.

To select more active ligands in each iteration, one can simply evaluate more ligands per iteration. While this does improve the Kendall-tau correlation and the rate of discovery of strong binders in the early iterations, increasing the number of randomly selected ligands evaluated to build the very first model significantly decreases the number of identified active compounds per number of evaluated ligands in the starting iteration (Figure S5).

Overall, the *uncertain* and *random* ligand selection sampling covers broadly the chemical library and provides a better overall description of the chemical space. However, to efficiently identify most potent binders, other strategies, e.g., *greedy* or *narrowing*, are preferred. As we are not primarily interested in an accurate description of medium and low affinity compounds, we can choose to sacrifice the accuracy of the general data set quantification and proceed with those strategies that are capable of best uncovering the most potent compounds.

#### 3.2.3 Active Learning

**Figure 3:**
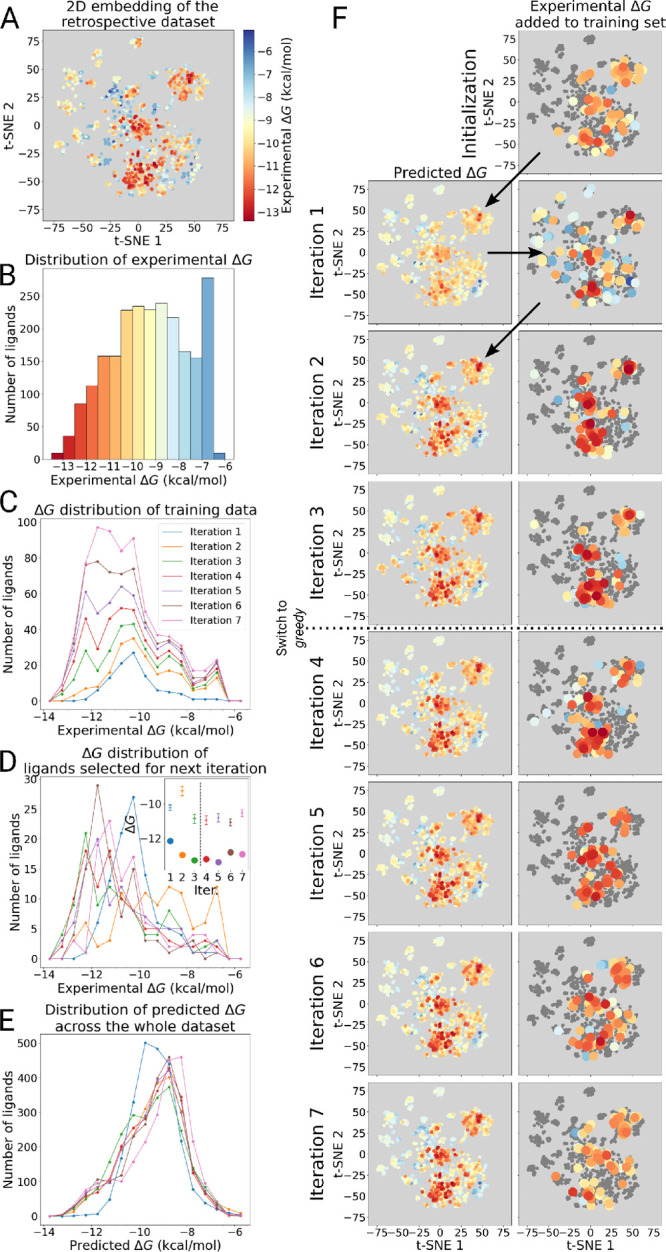
Characterization of the retrospective data set and progression of one repeat of the active learning protocol using the *narrowing* selection rule to pick 100 new ligands per iteration. (A) T-SNE embedding into 2D space based on Tanimoto similarity coefficients shows three clusters of strongly binding ligands. (B) Distribution of the experimental binding free energies shows the overall number of such strong binders to be small. As the protocol progresses, the number of identified high affinity ligands increases (C), as at each iteration 100 new ligands are selected (D) to be added to the training set. The inset in panel (D) shows distribution means (with 95% confidence intervals) and strongest binders found at each iteration. After the first iteration, the distribution of binding free energies predicted using models based on the 2D_3D representation (E) remains stable. Ligands selected at each iteration in one repeat of the protocol (F, left) and the neural network predictions for the binding free energies of all the ligands in the same iteration (F, right).

As the ligand encoding and selection strategies have been explored, we further illustrate the overall active learning based chemical space exploration cycle using the same experimentally characterized set of PDE2 inhibitors (Figure 3). Here, we relied on the *narrowing* protocol of ligand selection and performed 6 iterations of model training and the subsequent binding affinity prediction. As the ligand library is analyzed retrospectively, we readily have access to the experimentally determined affinities (Figure 3A,B).

Analyzing the chemical space reveals three clusters of high affinity binders, yet the overall number of such ligands is low. The aim of the active learning procedure is to identify these potent molecules.

We start with a weighted (to reduce the chances of very similar ligands being chosen) random selection of 100 ligands from our library and retrieve their binding free energies. Following the *narrowing* selection approach, the active learning protocol broadly explores the chemical landscape due to disagreements between models based on different ligand representations in the first two iterations. By iteration 2, the top models agree that the best ligands reside in the same three clusters we identified (Figure 3A).

From the third iteration the *narrowing* selection rule behaves exactly as the *greedy* protocol. The procedure switches to training an ensemble of five models only on the 2D_3D representation. The next 100 ligands are selected based on the mean prediction of these five models, allowing for some cancellation of errors in regions of insufficient training data as a result of this ensemble approach. As active learning continues, it focuses on the three high affinity regions and selects ligands with lower experimental binding free energies than initially (Figure 3D). The training sets also become progressively biased toward strongly binding ligands with each iteration (Figure 3C). Despite this, the distribution of predicted binding free energies does not change much following the initial iteration (Figure 3E).

Eventually, though, after the majority of the strongly binding ligands have already been identified and added to the training set, their pool is exhausted. With the decrease in number of strong yet unidentified binders, the models begin to backfill the training set with weaker binding ligands: the strongest identified binders at iterations 5 and 6 do not outcompete the best binders identified earlier (inset in panel D of Figure 5). As the probability of finding even higher affinity ligands now drops with each iteration while the computational cost remains the same, about 5 iterations appears to be optimal for halting the active learning process.

### 3.3 Part II: Prospective Ligand Optimization

Having verified and identified the limits of adaptive learning protocols in a retrospective analysis (Part I), we have applied this approach prospectively to identify novel high affinity PDE2 inhibitors. In Part II, the alchemical free energy calculations were used as an oracle in the active learning cycle (Figure 1).

#### 3.3.1 Library Generation and Alchemical Oracle

**Figure 4:**
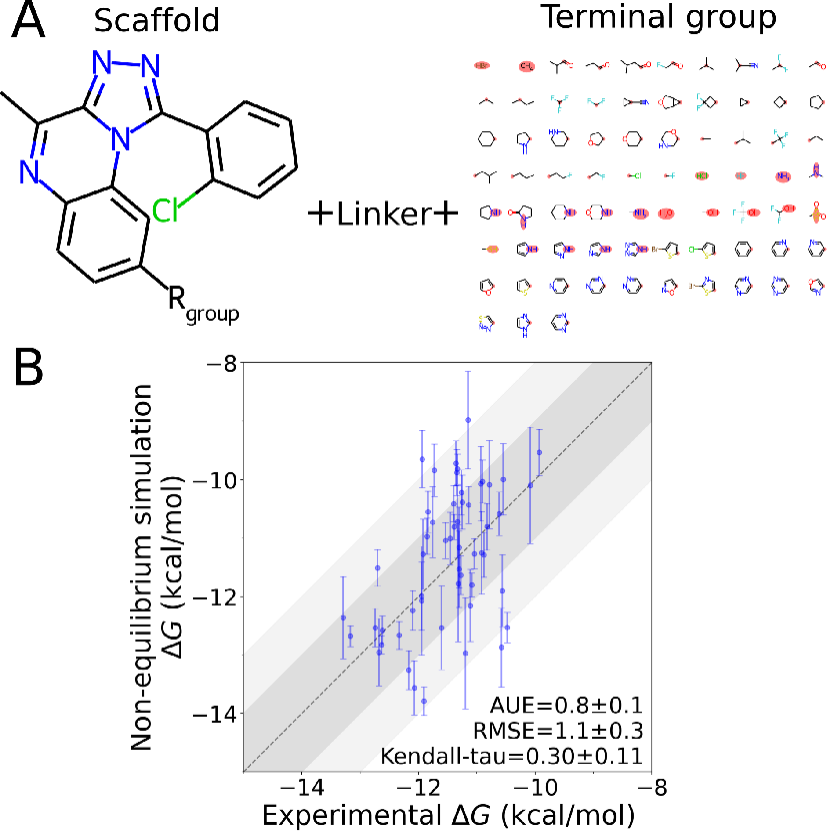
Ligand library construction and validation of alchemical free energy calculations. (A) For the library construction, one ligand scaffold was selected from the data set investigated in the Part I. The scaffold was combined with a set of linkers (Figure S6) and termini, using the chemical groups marked in red to form the covalent bonds. (B) Validation of binding free energies obtained with nonequilibrium free energy calculations against experimental results.

For the search of potent inhibitors, we constructed a custom library of 34 114 compounds. For that, we selected one scaffold from the data set analyzed in Part I as a core and attached varying R-groups at a common position. Such library design based on a common scaffold ensures that relative binding free energies can be calculated accurately, thus providing reliable decisions by the oracle in the AL cycle. Each R-group was composed of functional groups present in the data set analyzed in the first part of the investigation. The R-groups comprised a linker attached to the core (Figure S6) decorated with up to three terminal groups (Figure 4A).

**Figure 5:**
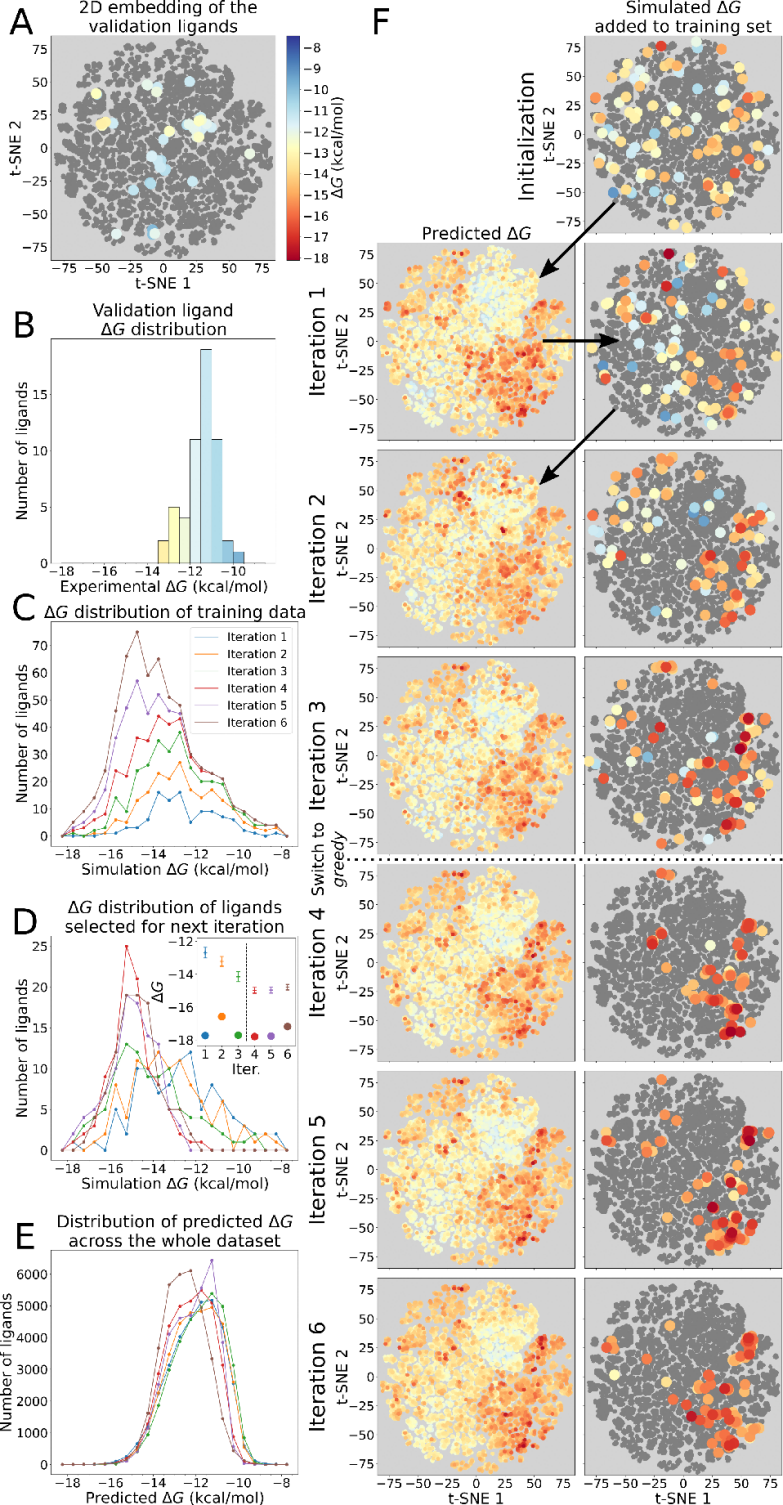
Progress of the active learning algorithm used with the prospective ligand library. Validation ligands highlighted inside the (A) t-SNE embedding^36^ for the prospective library and (B) the distribution of their experimental binding free energies. (C) Distribution of the calculated free energies of the training ligands, and (D) those selected for evaluation of binding free energies at each iteration. The inset in panel (D) shows distribution means (with 95% confidence intervals) and strongest binders found at each iteration. (E) Distribution of predicted free energies over the whole prospective ligand library at each iteration. Calculated binding free energies of the ligands selected to be added to the training set at each iteration and free energies predicted by the models at those iterations all displayed on a t-SNE embedding of the whole prospective ligand library. Arrows indicate progress of the active learning protocol.

Since in the prospective analysis experimental affinity measurements were not available, we computed binding free energies of the ligands selected by the protocol of MD-based alchemical simulations and trained the neural networks on the calculated affinities. The alchemical approach yields accurate predictions of free energies, which we benchmarked on a subset of ligands from the experimental library from Part I that share the same scaffold as the prospective library (55 molecules). The root mean squared error (RMSE) of the computed values compared to the experimental measurement was 1.1 ± 0.3 kcal/mol (Figure 4B). In addition to the computational error, experimental measurements also have an associated uncertainty. For a similar set of PDE2 inhibitors a standard deviation for measurements of a bioactivity assay is reported to be 0.3 kcal/mol.^60^ This, however, likely represents a lower bound of the true experimental error, as repeated pIC_50_ measurements for the same compound and protein show standard deviations of ∼0.9 kcal/mol.^61^ All in all, propagating uncertainties from computation and experiment, we estimate the difference between the computed and measured Δ*G* to have an associated error of 1.1–1.4 kcal/mol. The benchmarked compounds together with the reference ligand (55 molecules total) also appear in the currently investigated prospective library and, so, are further used for validation of model predictions (Figure 5A,B).

#### 3.3.2 Active Learning of the Prospective Data Set

The behavior of the active learning protocol trained on MD based free energies was similar to the models trained on experimental free energies in the retrospective analysis in Part I (Figure 3). The protocol initially explored the chemical space to find promising high affinity regions (Figure 5F). After the switch to using the best predicted ligands, *greedy* selection, from models based only on the 2D_3D ligand representation in the third iteration, the protocol predominantly focused on one region of chemical space. In this subspace, a substitution of a benzene ring directly bound to the scaffold was preferred, and probing molecules with this chemistry returned higher affinity hits (Figure 5C,D). Finally, at the sixth iteration, the number of found high affinity ligands dropped, indicating their pool was likely exhausted. Therefore, the protocol was stopped at this point.

**Figure 6:**
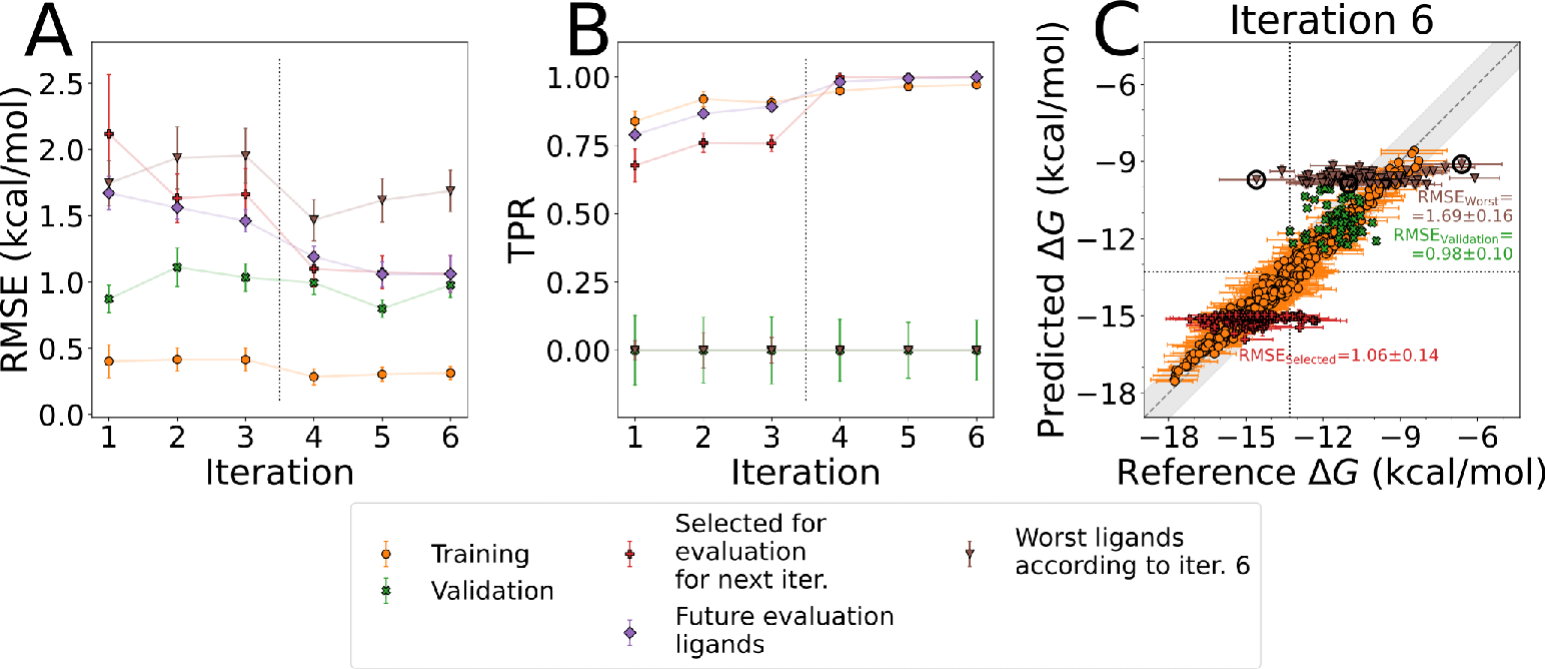
Metrics of model accuracy (A, B) in active learning on the prospective ligand library and a comparison of the predicted and reference binding free energies used in its sixth iteration (C). Predictions for validation ligands are compared to experimental binding affinities, while for all other ligands free energies from simulations are used as the reference. Vertical dotted lines in (A) and (B) represent the switch from using models from multiple ligand representations to a single one. For regression models, a true positive rate can only be determined relative to a threshold. Here we use a threshold of −13.29 kcal/mol, equal to the experimental binding free energy of the best binding validation ligand and depicted as dotted lines in panel (C). The gray region indicates absolute unsigned errors of up to 1 kcal/mol. Error bars represent standard errors of the mean and for RMSE and TPR are determined via bootstrapping.

The RMSEs for both the experimental validation ligands (green curve) and those yet to be evaluated via simulations (purple curve) show low RMSE values, by the end of the learning cycle reaching 1.00 ± 0.09 and 1.19 ± 0.08 kcal/mol, respectively (Figure 6A). These error magnitudes are already on par with those expected from the reference MD simulations. Furthermore, as the iterations progress and more ligands are added to the training set, model accuracy increases. This can be seen from the decrease in RMSE for ligands that will be evaluated for subsequent iterations (Figure 6A). The prediction accuracy for validation ligands (molecules with the experimentally measured affinities) does not change much with every iteration, yet a low RMSE value of ∼1 kcal/mol is retained. As the ligands from the validation set all have fairly low binding affinities, few chemically similar molecules are added to the training set, leading to little opportunity for improvement in those regions of chemical space.

Both ligands from the validation set and those selected at each iteration have narrow dynamic ranges of predicted binding free energies (Figure 6C). Combined with the remaining model errors, this leads to low correlations between the predicted and reference free energies, when evaluated within those ligand sets. Additionally, the predicted free energies of the strongest binders selected at each iteration are often underestimated (Figure 6C). Nevertheless, the models are still able to distinguish strongly and weakly binding ligands, reaching near perfect true positive rates for the yet unmeasured ligands in later iterations (Figure 6B). Furthermore, every iteration provides further enrichment of high affinity ligands in the training set.

#### 3.3.3 Strongest Binders

**Figure 7:**
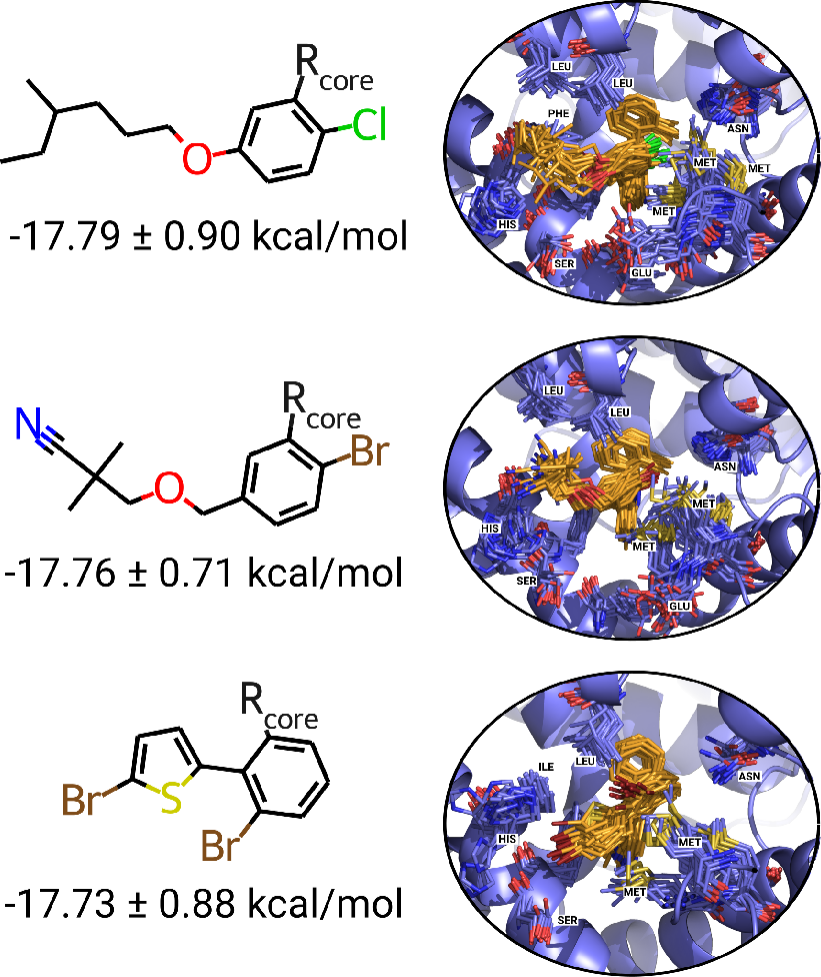
Strongest binding ligands found, their calculated binding free energies, and binding poses sampled with molecular dynamics.

We terminate the active learning procedure for the prospective data set after six iterations and further explore the identified highest affinity binders. The chemical space exploration yields several ligands with computed binding free energies below −17 kcal/mol that have been found, while the lowest binding free energy of the experimentally known ligands with this scaffold was above −14 kcal/mol (Figure 5B). The best binding ligands found in the prospective library are depicted in Figure 7. All the high affinity ligands show a similar pattern of substitution at the same functional group. A benzene ring serves as a linker to the scaffold, and two groups decorate the ring: a single halogen atom at the 4 or 5 ring position and a longer flexible hydrophobic group that sometimes also contains an electronegative atom.

For the strongest binders, the increase in affinity is provided by the interactions with hydrophobic residues in the vicinity. We analyzed the most frequent contacts in the simulations for the protein ligand complexes in Figure 7 and observed that the optimized functional group mostly interacted with leucine, methionine, and histidine side chains. The halogen atoms and ether group also allow for favorable contacts with serine and glutamate.

## 4 Discussion

### 4.1 Ligand Selection

The nature of the chemical space exploration by means of active learning (Figure 1) can be strongly influenced by the strategy of ligand selection for ML model training. The *random* and *uncertain* strategies provided a decent general description of the compound library, yet for a task of lead optimization, one might prefer to obtain a better description of the most potent binders. The *greedy* and *narrowing* ligand selection approaches rapidly find strongly binding ligands, at the cost of better describing high affinity ligands than low affinity ones. For example, the last iteration of the active learning protocol on the prospective library predicts binding free energies for the ligands with the strongest predicted binding at an RMSE of 1.06 ± 0.14 kcal/mol, while for the weakest predicted binders the RMSE is 1.69 ± 0.16 kcal/mol (Figure 6C). This comes about due to the much smaller number of training ligands in the low affinity regions (Figures 3C and 5C). Still, free energy calculations show only two of the 94 worst predicted binders that were simulated to have stronger affinities than the validation ligands do in experiments, indicating a low false negative rate for such predictions.

Although in the prospective investigation of this work (Part II) we used the *narrowing* selection strategy, in practical terms computing many descriptors required for the first iterations may be computationally expensive. Retrospectively, we have also probed a simple variant of such a *narrowing* approach, where for the first three iterations ligands are selected randomly and afterward the procedure switches into the *greedy* mode. Such a *random2greedy* strategy finds the strongest binders slower; however, the final results in the end offer a good balance between the identified high affinity ligands and the overall accurate description of the chemical library (Figure S8).

While the selection strategy and ligand encoding have a strong effect on the accuracy of the models, the AL procedure appears to be robust with regard to the initial conditions. When starting with disparate ligand selections from localized chemically similar molecule clusters, the final models converge to comparable final predictions (Figure S9). This is encouraging, as in practical applications it may be convenient to initialize the AL cycle from the chemically similar congeneric series of compounds with experimentally readily measured affinities. The recall of the AL approach appears to be robust and independent of the exact number of actives in the set (Figure S10), thus making it an appealing candidate for large scale prospective studies.

### 4.2 Predicted Weak Binders

While we have already inspected the chemistry of the predicted high affinity compounds (Figure 7), it is also interesting to understand what molecules were identified by the models as particularly weak binders. It appears that, in the prospective study (Part II), a large fraction of molecules predicted as low affinity binders contained sulfonyl groups (Figure 6C). Although alchemical calculations did not find any of the sulfonyl containing ligands to be strong binders, some did reach medium binding affinities of −15 kcal/mol. Thus, sulfonyl should not necessarily be a disqualifying factor for ligand affinity to PDE2. Yet, why does this chemical group dominate the predicted low affinity ligands?

### 4.3 Molecular Composition Bias

We identified this tendency to be largely due to use of inverse-frequency weighting,^62^ which scales the impact of the ligands on the model based on the inverse probability of their reference free energy in the training set (SI Figure S7). While this technique was intended to compensate for bias due to increasing number of active compounds in the *greedy* and *narrowing* selections, it also has a side-effect causing ligands from poorly sampled regions of the Δ*G* spectrum to have a larger impact on the loss function. Inverse-frequency weighting does not significantly change the free energy distribution of sulfonyl containing ligands. Instead it makes other ligands less likely to be classified as weak binders. Therefore, while compensating for bias due to the free energy distribution of the training set, inverse-frequency weighting also exposes bias due to ligand composition.

One approach to control the molecular composition bias in ligand selection for model training is to use ligand representations that do not rely directly on molecular composition but instead on physical interactions between the ligand and the protein. Examples of such representations are interaction energies computed using molecular mechanics force fields (MDenerg) or protein–ligand interaction fingerprints (PLEC). In the current study, however, none of these representations were able to outperform simple 2D_3D ligand based descriptors. More generally, training models on ligand-only information leads to memorization of ligand features,^63^ even across different host proteins.^64^ A wishful thought is for the model to learn the underlying physics by providing protein–ligand interaction descriptors as input. In practice, though, doing so hardly improves the accuracies of the models in question,^64^ at least not without extensive sampling of the model applicability space in the training set.^65^ Furthermore, many lead optimization data sets such as that used here exhibit 2D bias as the molecules were often designed and synthesized iteratively based on underlying 2D chemical similarity principles.

### 4.4 Further Improvements for the AL in Chemical Space Exploration

Drawing ligand selections from a variety of models built on different representations was expected to help active learning more uniformly sample the chemical space in the early iterations. However, since only the best predicted binders were selected from every model, the *narrowing* scheme performs similarly to the simple *greedy* protocol, which relies on a single ligand representation (Figure 2). Both approaches are efficient at quickly exploring a narrow branch of the chemical space to identify potent binders. Interestingly, the *uncertainty*-driven protocol performs well in an overall description of the chemical library. It sacrifices the ability to quickly identify high affinity binders, but includes a broad range of ligands in the training set, thus providing a more accurate global description of the ligand set. For future investigations, it might be a promising avenue to combine *uncertain* and *greedy* protocols either into a *narrowing*-style scheme, where the first few iterations are handled with the *uncertain* selection rule and later iterations by the *greedy* one, or a modified *mixed* scheme, where the ratio of the most uncertain to most strongly binding predictions is changed at each iteration to smoothly transition from the *uncertain* to *greedy* selection during active learning.

Reliance on MD calculations for the ground truth to train the active learning models allows one to perform ligand optimization completely *in silico*. While docking would be a faster alternative, MD calculations provide a much more accurate measure of the binding free energy, one that explicitly takes entropic contributions into account. Nevertheless, MD still suffers biases due to force field errors and uncertainties from incomplete sampling of phase space. While this approach can eliminate the need for effort intensive ligand synthesis and experimental affinity measurements during the course of the ligand optimization process, affinities of the final ligand selections still need to be experimentally validated.

## 5 Conclusion

In the current work we have developed an approach for lead optimization combining active learning and alchemical free energy calculations. In the first part of the investigation, we calibrated the machine learning procedure on a large data set of PDE2 inhibitors using experimentally measured affinities. Subsequently, in the second part of the work we have used the approach in a prospective manner relying on the calculated binding affinities as an oracle in the active learning cycle.

All in all, we demonstrate that the active learning approach can be combined with alchemical free energy calculations for an efficient chemical space exploration, navigating toward high affinity binders. The iterative training of machine learning models on an increasing amount of data allows the number of compounds to be evaluated with the more computationally expensive methods to be significantly reduced. An active learning procedure can be tuned to capture different characteristics of the chemical library: in the current work we demonstrate how to quickly identify the most potent binders, while sacrificing the quality of the overall description of the chemical library. This objective, however, can be altered by the particular choices within the active learning loop.

## Supporting Information Available

Agreement in selecting the top ligands between multiple repeats of the *greedy* protocol for different representations, convergence of predicted binding free energies, agreement in selecting the top ligands between multiple repeats using different selection rules with the 2D_3D representation, model accuracies, linker fragments used to construct the library, comparison of model predictions, accuracy comparisons of different ligand selection strategies, performance of the *narrowing* selection procedure, percentage of the strongest identified binders using the *narrowing* selection procedure in retrospectively analyzed data set with varying number of active compounds, meta-parameters, and components of the 2D_3D representation (PDF)
