# Supplementary material for: Chemical Space Exploration with Active Learning and Alchemical Free Energies
Source: J Chem Theory Comput. 2022 Sep 23;18(10):6259–70. doi: 10.1021/acs.jctc.2c00752 (PMC9558370; doi:10.1021/acs.jctc.2c00752)
Supplement: Supplementary file 1 — ct2c00752_si_001.pdf [file ct2c00752_si_001.pdf]

Supporting Information: Chemical Space Exploration with Active Learning and Alchemical Free Energies

Yuriy Khalak[1], Gary Tresadern[2], David F. Hahn[2], Bert L. de Groot[1], and Vytautas Gapsys[1,*]

[1] Computational Biomolecular Dynamics Group, Department of Theoretical and Computational Biophysics, Max Planck Institute for Multidisciplinary Sciences, Am Fassberg 11, D-37077, Göttingen, Germany.
[2] Computational Chemistry, Janssen Research & Development, Janssen Pharmaceutica N. V., Turnhoutseweg 30, 2340, Beerse, Belgium.
[*] vgapsys@gwdg.de

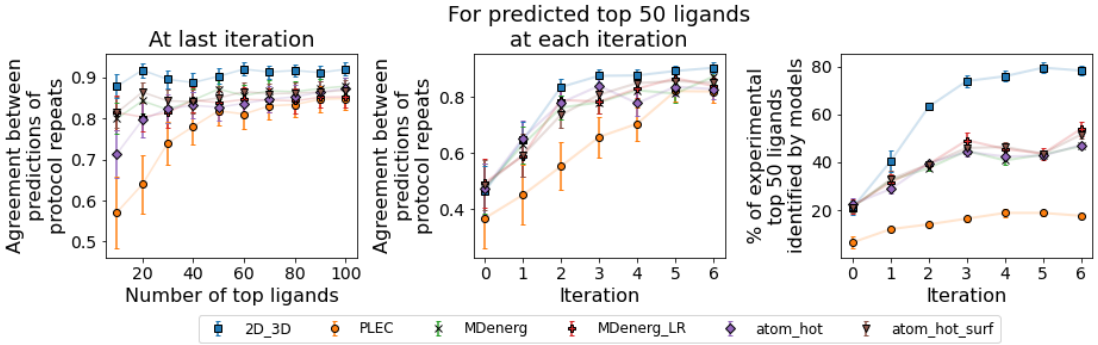

Figure S1: Agreement in selecting the top ligands between multiple repeats of the *greedy* protocol for different representations. Agreement is the number of common top N ligands between two repeats divided by N. Uncertainties were estimated via bootstrap of repeat pairs. The 2D_3D representation (composed of the chemoinformatic features) results in the most consistent predictions.

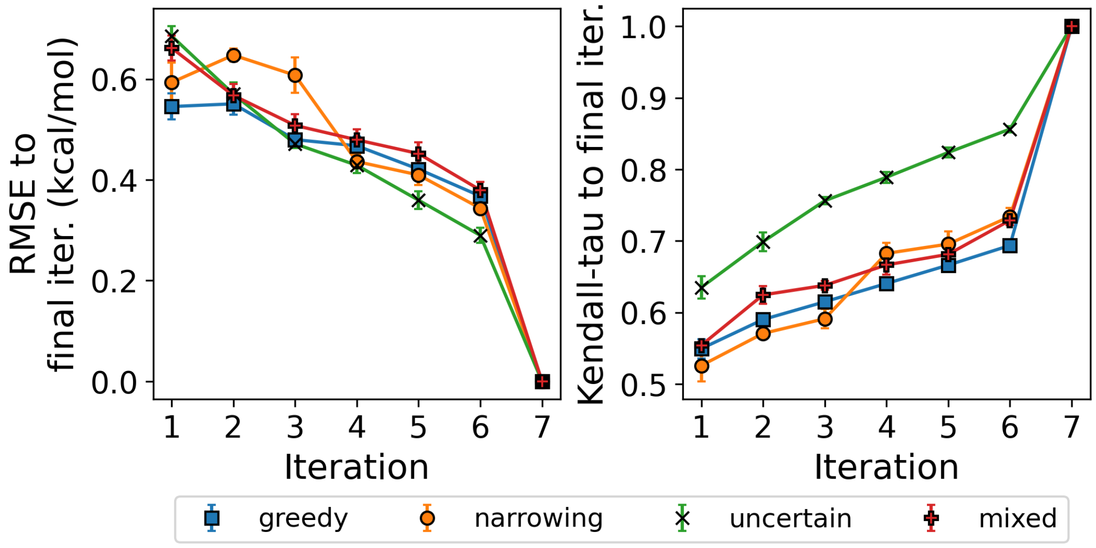

Figure S2: Convergence of predicted binding free energies towards those of the 7th (final) iteration evaluated using ligands not part of the final iteration's training set. Uncertainties are standard errors of the mean across five protocol repeats. The *uncertain* selection strategy converges faster than others.

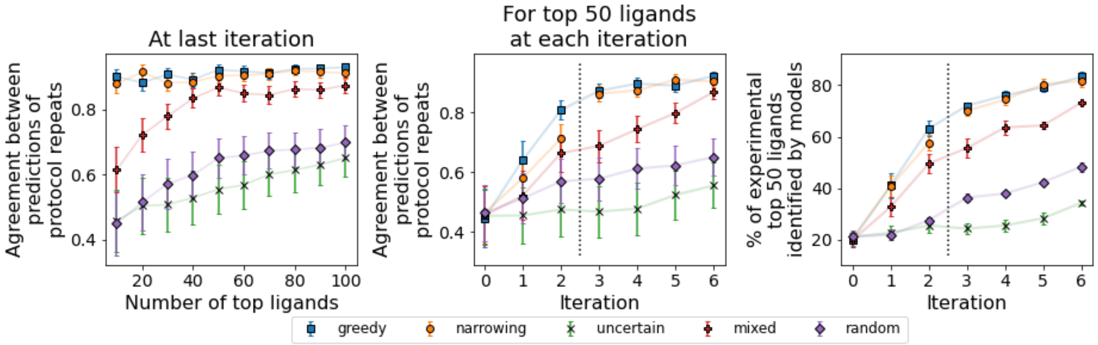

Figure S3: Agreement in selecting the top ligands between multiple repeats using different selection rules with the 2D_3D representation. Agreement is the number of common top N ligands between two repeats divided by N. Uncertainties were estimated via bootstrap of repeat pairs. *Greedy* and *narrowing* selection rules are more consistent in their selection of top ligands throughout the protocol.

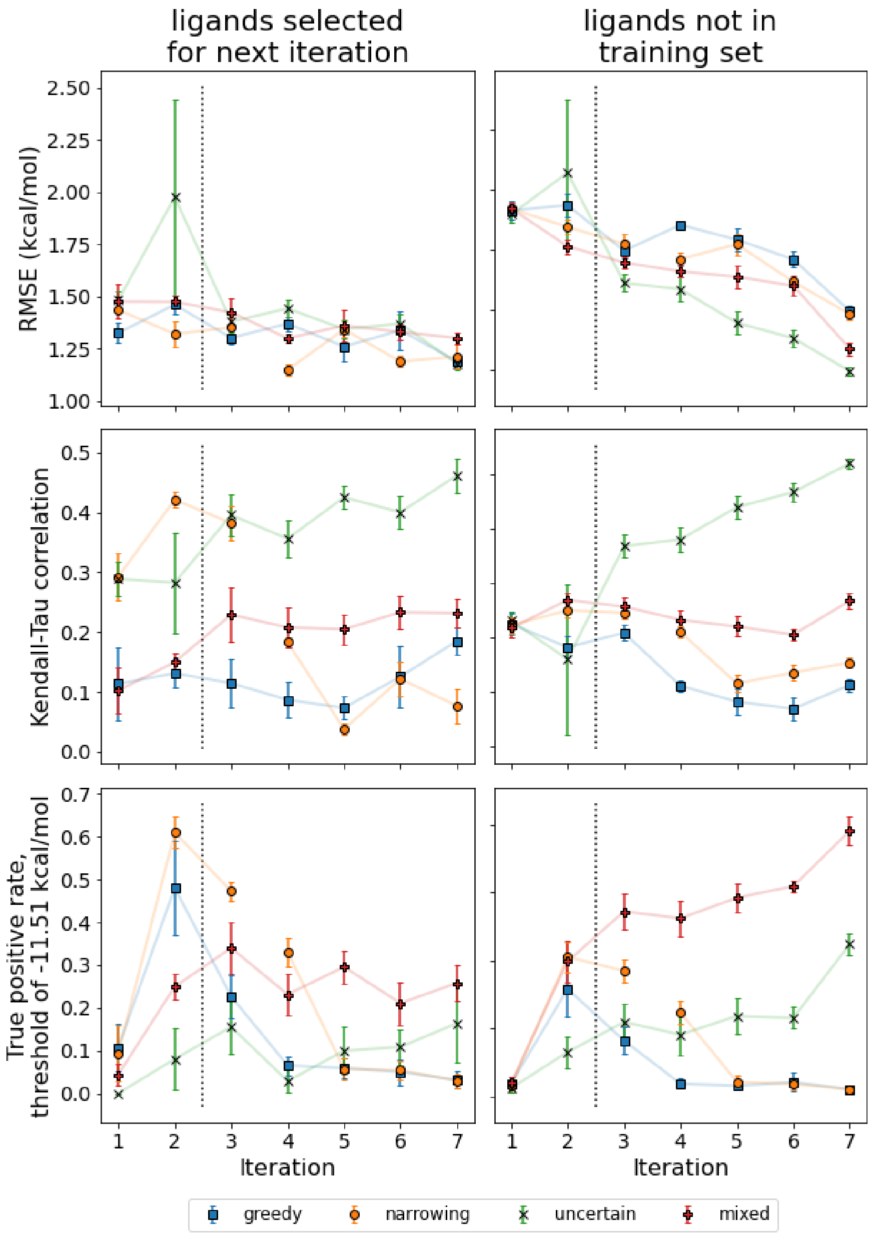

Figure S4: Model accuracies for ligands not in the training set and the subset of them selected for evaluation and addition to the training set at each iteration. While errors in the binding free energies are low, the faster converging approaches have incorrect ordering of the selected ligands at each iteration, resulting in low true positives rates.

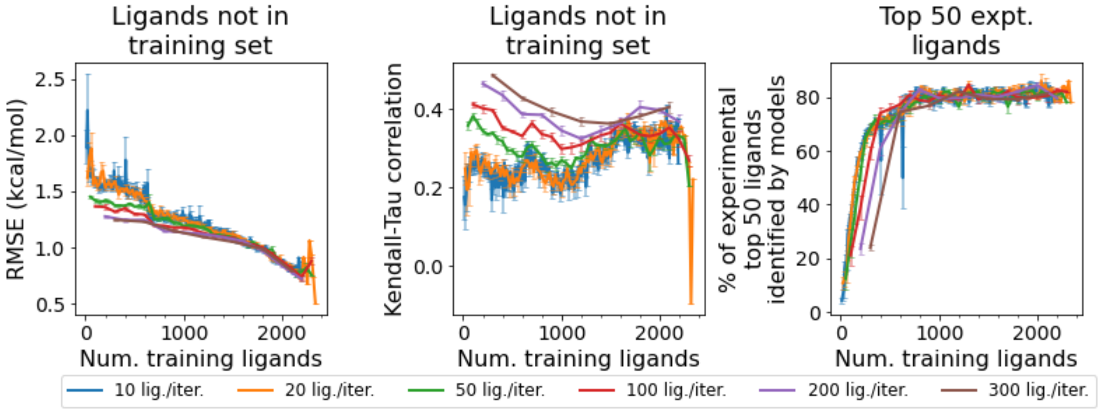

Figure S5: Model accuracies and discovery of strongest binders with different numbers of ligands evaluated at each active learning iteration. Error bars represent standard errors of the mean. While initial iterations each depict means over five repeats of the active learning protocol, fewer repeats are present at later iterations, especially for small numbers of ligands per iteration, hence the increasing noise.

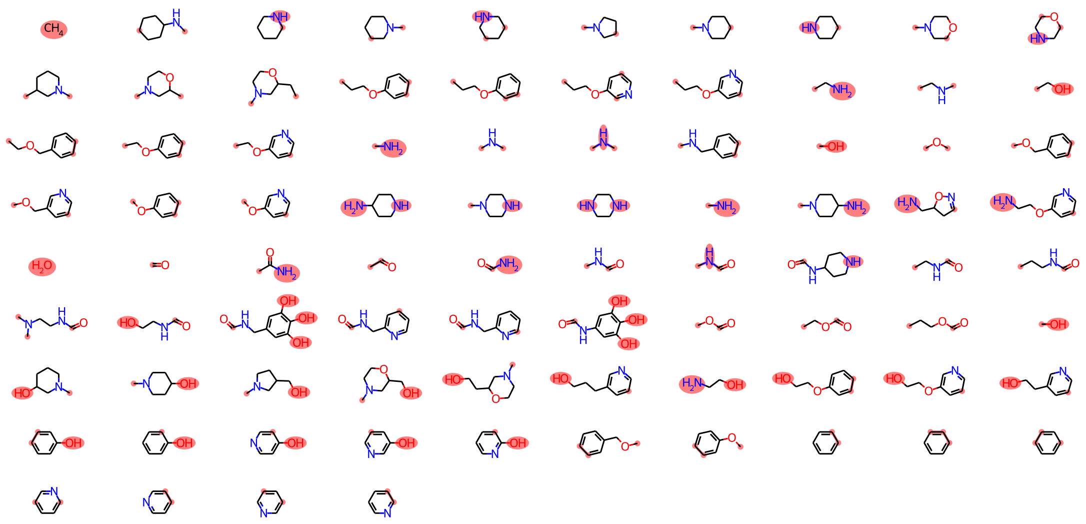

Figure S6: Linker fragments used to construct the library. Red highlights illustrate attachment points for the scaffold and termini.

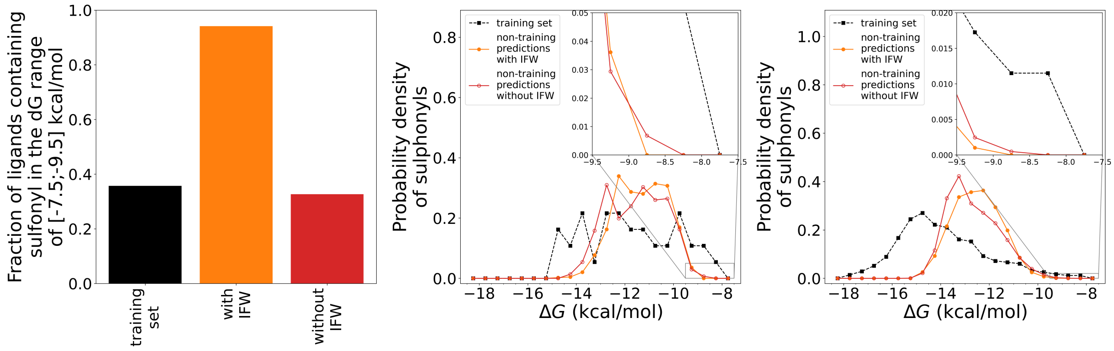

Figure S7: Comparison of model predictions for sulfonyl containing ligands with and without inverse-frequency-weighting (IFW). IFW makes such ligands over-represented among the low affinity predictions without changing the distribution of their free energies.

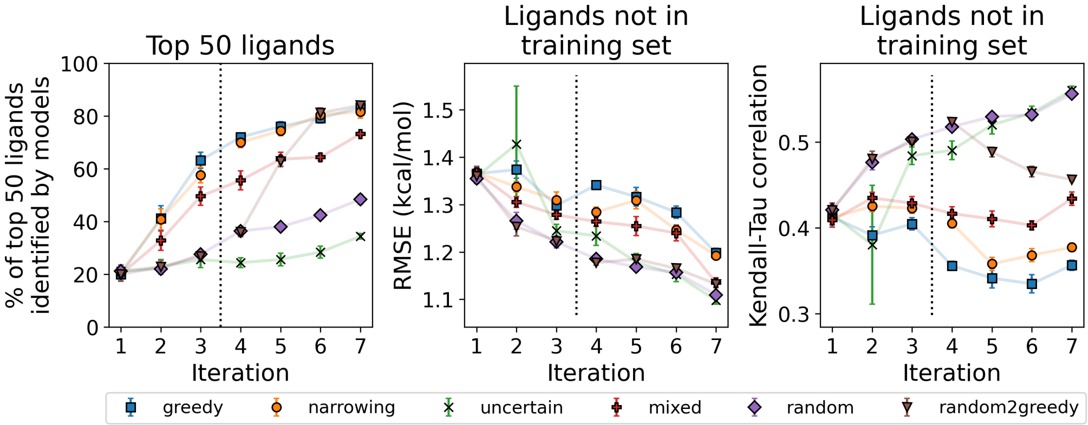

Figure S8: Accuracy comparisons of different ligand selection strategies. In this comparison *random2greedy* selection strategy is added. In this strategy, ligands for the first three iterations are selected randomly. Afterwards, the procedure switches into the *greedy* selection mode.

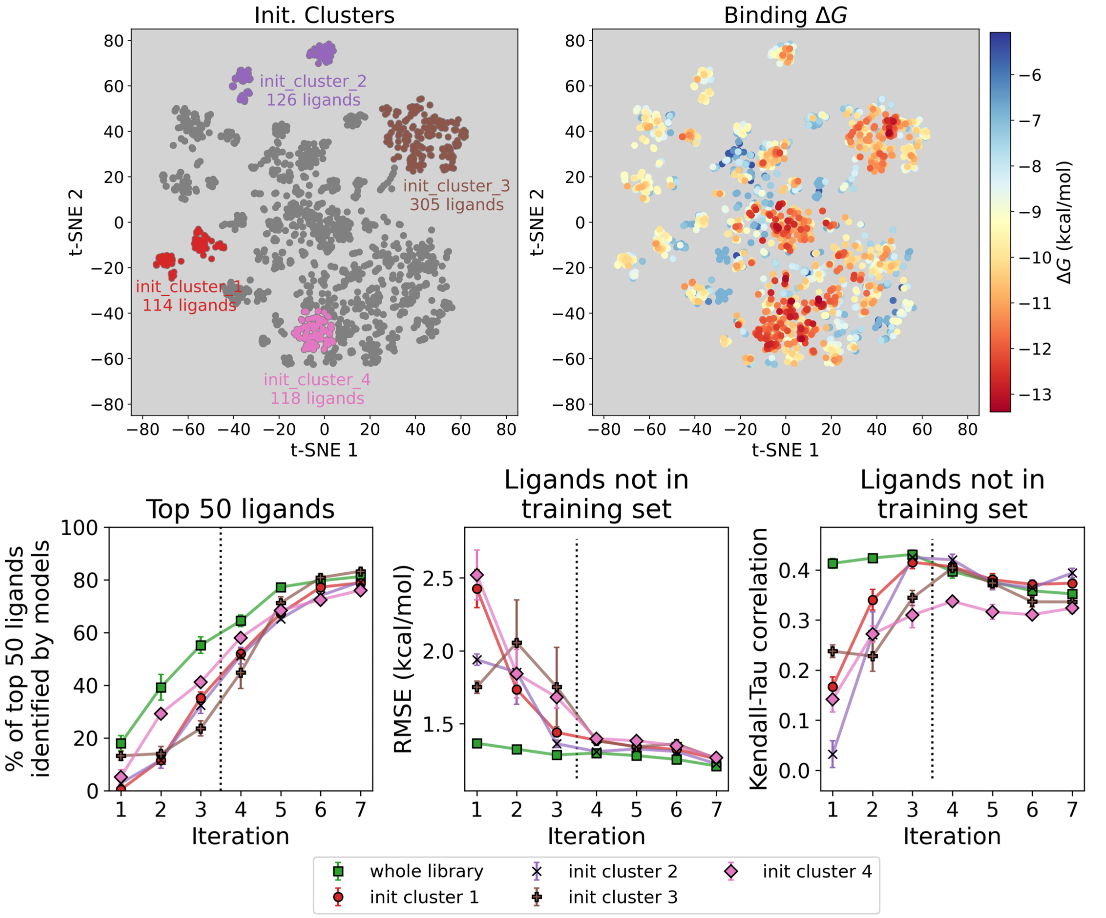

Figure S9: Performance of the *narrowing* selection procedure when initialized with different ligand selections. Initialization was done by randomly selecting 100 ligands from each cluster or from the whole library.

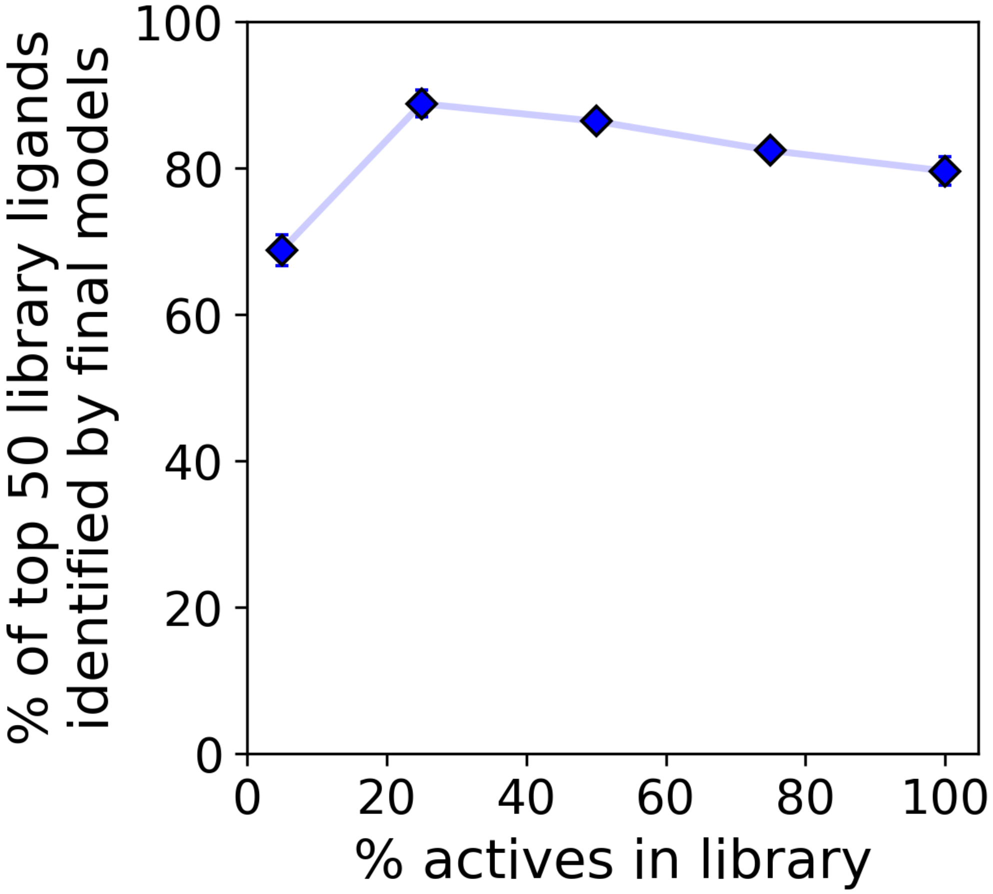

Figure S10: Percentage of the strongest identified binders using *narrowing* selection procedure in retrospectively analyzed data set with varying number of active compounds. These data sets were constructed by removing a fraction of randomly selected active ligands ($\Delta G < -11$ kcal/mol) from the full retrospective data set before applying the active learning protocol.

Table S1: Meta-parameters used for training with the prospective dataset. The first three iterations used 2D_3D, PLEC, atom_hot, and atom_hot_surf representations, two copies each of MDenerg and MDenerg_LR representations, as well as R-group only versions of all of the above representations.

Iteration	Epochs	Hidden layers		ΔG normalisation		Feature	Ligand
		depth	width	scale	bias	importance cutoff	encoding
1	2000	2	10	0.6515	9.512	none	multiple
2	20000	2	10	0.6515	9.512	none	multiple
3	20000	2	10	0.6515	9.512	none	multiple
4	20000	2	300	0.6515	9.512	0.02	2D_3D + PLEC
5	20000	3	20	0.6515	9.512	none	2D_3D
6	20000	3	20	1.0	13.04	none	2D_3D

Table S2: Meta-parameters used for training with the retrospective dataset. Ligand encoding varies depending on the experiment. In experiments using the *narrowing* selection (eg. Figure 3), the first three iterations used 2D_3D, PLEC, atom_hot, and atom_hot_surf representations as well as two copies each of MDenerg and MDenerg_LR representations.

Iteration	Epochs	Hidden layers		ΔG normalisation		Feature	Ligand encoding
		depth	width	scale	bias	importance cutoff	for *narrowing*
1	2000	2	300	0.6515	9.512	none	multiple
2	20000	2	10	0.6515	9.512	none	multiple
3	20000	2	10	0.6515	9.512	none	multiple
4	20000	3	20	0.6515	9.512	none	2D_3D
5	20000	3	20	0.6515	9.512	none	2D_3D
6	20000	3	20	0.6515	9.512	none	2D_3D
7	20000	3	20	0.6515	9.512	none	2D_3D

Table S3: Components of the 2D_3D representation. Common RDKit descriptors include constitutional and electric electrotopological descriptors some of which also appear in other feature groups and can be listed via rdkit.Chem.Descriptors._descList. Pharmacophore feature maps look for common chemical groups in the same positions as in ligands from 4d08[2], 4d09[2], 4htx[16], 6cyd[12], 6ezf[10] and 13 unpublished PDE2 crystal structures (shared with us by Janssen Research & Development). Features for EState, PEOE, SMR, and SlogP are encoded on the molecular van der Waals surface to form fingerprints using the VSA[6] approach.

Feature Group or Fingerprint	Number of Features
Common RDKit descriptors	208
Graph descriptors	16
Weiner index[14]	1
MACCS[3] fingerprint	167
MORSE[13] fingerprint	224
GETAWAY[13] fingerprint	273
BCUT[9]	8
pharmacophore feature maps[7]	358
molecular quantum numbers (MNQs)[8]	42
2D autocorelations[13]	192
3D autocorelations[13]	80
Weighted holistic invariant molecular (WHIM) fingerprint[13]	114
Radial density function (RDF)[13]	210
Ultrafast shape recognition (USR)[1]	12
Ultrafast shape recognition with pharmacophoric constraints (USRCAT)[11]	60
electrotopological state (EState)[5]	158
partial charges (PEOE)[4]	14
molar refractivity (SMR)[15]	10
hydrophobicity (SlogP)[15]	12

References

[1] Pedro J. Ballester and W. Graham Richards. Ultrafast shape recognition to search compound databases for similar molecular shapes. *J. Comput. Chem.*, 28(10):1711–1723, 2007. ISSN 1096-987X. doi: 10.1002/jcc.20681.

[2] Peter Buijnsters, Meri De Angelis, Xavier Langlois, Frederik J. R. Rombouts, Wendy Sanderson, Gary Tresadern, Alison Ritchie, Andrés A. Trabanco, Greet VanHoof, Yves Van Roosbroeck, and José-Ignacio Andrés. Structure-Based Design of a Potent, Selective, and Brain Penetrating PDE2 Inhibitor with Demonstrated Target Engagement. *ACS Med. Chem. Lett.*, 5(9):1049–1053, September 2014. ISSN 1948-5875, 1948-5875. doi: 10.1021/ml500262u.

[3] Joseph L. Durant, Burton A. Leland, Douglas R. Henry, and James G. Nourse. Reoptimization of MDL Keys for Use in Drug Discovery. *J. Chem. Inf. Comput. Sci.*, 42(6):1273–1280, November 2002. ISSN 0095-2338. doi: 10.1021/ci010132r.

[4] Johann Gasteiger and Mario Marsili. Iterative partial equalization of orbital electronegativity—a rapid access to atomic charges. *Tetrahedron*, 36(22):3219–3228, January 1980. ISSN 0040-4020. doi: 10.1016/0040-4020(80)80168-2.

[5] Lemont B. Kier and Lowell H. Hall. *Molecular Structure Description: The Electrotopological State*. Elsevier Science, May 1999. ISBN 978-0-12-406555-0.

[6] Paul Labute. A widely applicable set of descriptors. *Journal of Molecular Graphics and Modelling*, 18(4):464–477, January 2000. ISSN 1093-3263. doi: 10.1016/S1093-3263(00)00068-1.

[7] Gregory A. Landrum, Julie E. Penzotti, and Santosh Putta. Feature-map vectors: A new class of informative descriptors for computational drug discovery. *J Comput Aided Mol Des*, 20(12):751–762, December 2006. ISSN 1573-4951. doi: 10.1007/s10822-006-9085-8.

[8] Kong T. Nguyen, Lorenz C. Blum, Ruud van Deursen, and Jean-Louis Reymond. Classification of Organic Molecules by Molecular Quantum Numbers. *ChemMedChem*, 4(11):1803–1805, November 2009. ISSN 1860-7179. doi: 10.1002/cmdc.200900317.

[9] Robert S. Pearlman and K. M. Smith. Novel Software Tools for Chemical Diversity. In Hugo Kubinyi, Gerd Folkers, and Yvonne C. Martin, editors, *3D QSAR in Drug Design*, pages 339–353. Springer Netherlands, Dordrecht, 2002. ISBN 978-0-7923-4790-3 978-0-306-46857-5. doi: 10.1007/0-306-46857-3_18.

[10] Laura Pérez-Benito, Henrik Keränen, Herman van Vlijmen, and Gary Tresadern. Predicting Binding Free Energies of PDE2 Inhibitors. The Difficulties of Protein Conformation. *Scientific Reports*, 8(1):4883, March 2018. ISSN 2045-2322. doi: 10.1038/s41598-018-23039-5.

[11] Adrian M Schreyer and Tom Blundell. USRCAT: Real-time ultrafast shape recognition with pharmacophoric constraints. *J Cheminform*, 4:27, November 2012. ISSN 1758-2946. doi: 10.1186/1758-2946-4-27.

[12] Shawn J. Stachel, Richard Berger, Ashley B. Nomland, Anthony T. Ginnetti, Daniel V. Paone, Deping Wang, Vanita Puri, Henry Lange, Jason Drott, Jun Lu, Jacob Marcus, Michael P. Dwyer, Sokreine Suon, Jason M. Uslaner, and Sean M. Smith. Structure-Guided

Design and Procognitive Assessment of a Potent and Selective Phosphodiesterase 2A Inhibitor. *ACS Med. Chem. Lett.*, 9(8):815–820, August 2018. ISSN 1948-5875, 1948-5875. doi: 10.1021/acsmedchemlett.8b00214.

[13] Roberto Todeschini and Viviana Consonni. Descriptors from Molecular Geometry. In *Handbook of Chemoinformatics*, chapter VIII.2, pages 1004–1033. John Wiley & Sons, Ltd, 2003. ISBN 978-3-527-61827-9. doi: 10.1002/9783527618279.ch37.

[14] Harry Wiener. Structural Determination of Paraffin Boiling Points. *J. Am. Chem. Soc.*, 69 (1):17–20, January 1947. ISSN 0002-7863, 1520-5126. doi: 10.1021/ja01193a005.

[15] Scott A. Wildman and Gordon M. Crippen. Prediction of Physicochemical Parameters by Atomic Contributions. *J. Chem. Inf. Comput. Sci.*, 39(5):868–873, September 1999. ISSN 0095-2338. doi: 10.1021/ci990307l.

[16] Jian Zhu, Qiqi Yang, Dandan Dai, and Qiang Huang. X-ray Crystal Structure of Phosphodiesterase 2 in Complex with a Highly Selective, Nanomolar Inhibitor Reveals a Binding-Induced Pocket Important for Selectivity. *J. Am. Chem. Soc.*, 135(32):11708–11711, August 2013. ISSN 0002-7863, 1520-5126. doi: 10.1021/ja404449g.
